# McKittrick–Wheelock syndrome presenting with presumed paraneoplastic syndrome extra-ocular muscle enlargement masquerading as thyroid eye disease

**DOI:** 10.1530/EDM-22-0386

**Published:** 2023-06-21

**Authors:** Waqar Ahmad, Matthew Hartley, Shweta Singh, Kenzo Motohashi, Su Ann Tee, Helen Dallal, Dariush Kamali, Christopher Matthews, Shafie Kamaruddin

**Affiliations:** 1 County Durham and Darlington NHS Foundation Trust, UK; 2 North Tees and Hartlepool Hospitals NHS Foundation Trust, UK; 3 Gateshead Health NHS Foundation Trust, UK

**Keywords:** Adult, Female, White, United Kingdom, Thyroid, Thyroid, Unique/unexpected symptoms or presentations of a disease, June, 2023

## Abstract

**Summary:**

Paraneoplastic syndromes (PS) are uncommon and are known to mimic other clinical entities, often carrying significant morbidity and mortality. The commonest cause of extra-ocular muscle enlargement (EOME) is thyroid eye disease (TED). Rarely, PS can cause EOME and masquerade as TED. We describe a 52-year-old female who presented with diarrhoea, acute kidney injury and electrolyte imbalance. An ophthalmic review identified right upper lid retraction. MRI orbits showed increased thickness of the inferior and medial recti bilaterally, presumed as TED. Whilst investigating her diarrhoea, imaging revealed a large rectosigmoid tumour which required surgical excision. In the context of electrolyte disturbance and acute kidney injury, a diagnosis of McKittrick–Wheelock syndrome (MWS) was made. Following successful surgery, electrolyte imbalance, diarrhoea and eyelid retraction improved. Repeat MRI orbits displayed complete resolution of EOME. To our knowledge, this is the first case of MWS presenting with PS-EOME masquerading as TED.

**Learning points:**

- McKittrick–Wheelock syndrome (MWS) is a rare disorder, although likely under-recognised, which is characterised by diarrhoea, dehydration and electrolyte depletion that results from a hypersecretory colorectal neoplasm.
- Definitive treatment of MWS involves the resection of the colorectal neoplasm.
- Bilateral ophthalmopathy that appears to be Graves’ ophthalmopathy on imaging, though clinical and biochemical evidence fails to identify a thyroid pathology, has been associated with malignancy on rare occasions. Such patients should be investigated for potential malignant causes of their ophthalmopathy.

## Background

The commonest cause of extra-ocular muscle enlargement (EOME) is thyroid eye disease (TED), seen in 95% of cases in one large case series (1). This case report highlights the importance of broadening the differential diagnoses when signs and symptoms that are typically due to a single pathology present with atypical features.

Paraneoplastic syndrome (PS)-related orbital inflammation and EOME can mimic TED, and while TED predominantly affects the inferior rectus and PS-EOME predominantly affects the superior rectus, radiographically it can be challenging to distinguish them (2). Furthermore, EOME on imaging can be associated with normal thyroid function and minimal or no clinical signs or symptoms suggestive of thyroid dysfunction. Importantly, in one particular case series, non-thyroid-related EOME carried a significant mortality of 25%. Therefore, in any case of atypical EOME, the clinician should suspect more sinister aetiology other than thyroid related alone (3).

McKittrick–Wheelock syndrome (MWS) is a rare disorder, characterised by diarrhoea, dehydration, and electrolyte depletion (4). It is caused by a hypersecretory villous adenoma or adenocarcinoma located in the distal colon. Herein, we report a unique case of MWS in a patient with an associated ophthalmopathy that initially masqueraded as TED before being diagnosed as a PS of her rectosigmoid tumour.

## Case presentation

A 52-year-old Caucasian female with no significant medical history was admitted to a medical ward with persistent diarrhoea and multiple pre-syncopal episodes. She denied abdominal pain, nausea and vomiting, weight loss or pyrexia. Her father had been diagnosed with colorectal carcinoma at 49 years of age. Initial biochemistry showed acute kidney injury associated with hyponatraemia and hypokalaemia. On examination, the patient was noted to have new eyelid height asymmetry and possible proptosis. There were no reported symptoms of diplopia, pain or red eye.

**Figure 1:**
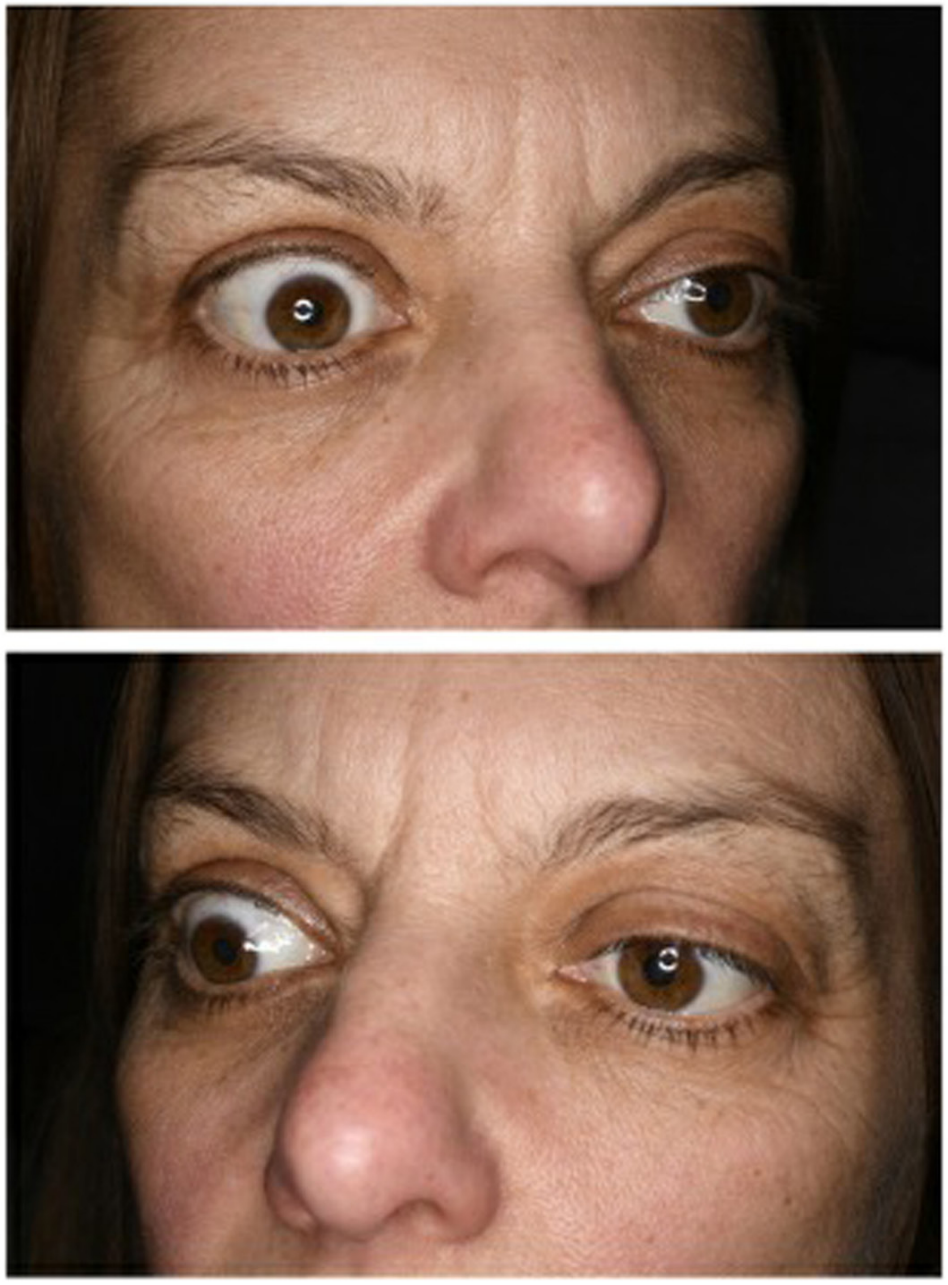
Right upper lid retraction on initial presentation.

She was examined in the eye clinic where the only positive finding was moderate right upper lid retraction; upper marginal reflex distance measured 7 mm right eye and 4 mm left eye (Fig. 1). The patient was unsure of the duration of her eyelid symptoms, but historical photographs revealed no asymmetry 6 months prior to presentation. There was no lid lag and no proptosis with Hertel’s exophthalmometer. A provisional diagnosis of TED was made; MRI was requested, and bloods were taken to assess thyroid function.

The patient was readmitted with recurring diarrhoea, acute kidney injury and deranged electrolytes (Table 1) for which she was thoroughly investigated. Random cortisol tests were repeatedly normal. Coeliac and autoimmune screens were normal (Table 1). Stool cultures were consistently reported as negative for microbial growth. Her aldosterone and renin levels were checked, and both came back raised which was consistent with secondary hyperaldosteronism in the presence of low potassium (Table 2).

**Table 1:** Celiac and autoimmune profile.

Investigations (serum)	February 2021
Anti-centromere antibodies	Negative
Anti-Ro & La antibodies	Negative
Anti-ribosomal P antibodies	Negative
Anti-RNP antibodies	Negative
Anti-chromatin antibodies	Negative
Anti-SLE 70 antibodies	Negative
Anti-DS DNA antibodies	Negative
Anti-smooth muscle antibodies	Negative
ANCA	Negative
Rheumatoid factor antibodies	Negative
Tissue transglutaminase antibodies	Negative
Serum immunoglobulin	Negative

**Table 2:** Hospital admission electrolytes and renal aldosterone levels.

Investigations	Normal range	February 2021	April 2021	October 2021
Sodium serum (mmol/L)	133–146	118	126	121
Potassium serum (mmol/L)	3.5–5.3	2.7	3.3	3.5
Urea serum (mmol/L)	2.5–7.8	41	18.9	37.1
Creatinine serum (µmol/L)	50–110	446	173	239
Chloride serum (mmol/L)	95–108	73	81	78
Aldosterone serum (pmol/L)	103–859			3600
Renin serum (mIU/L)	<59.7			>500

ANCA, antineutrophil cytoplasmic antibodies.

**Figure 2:**
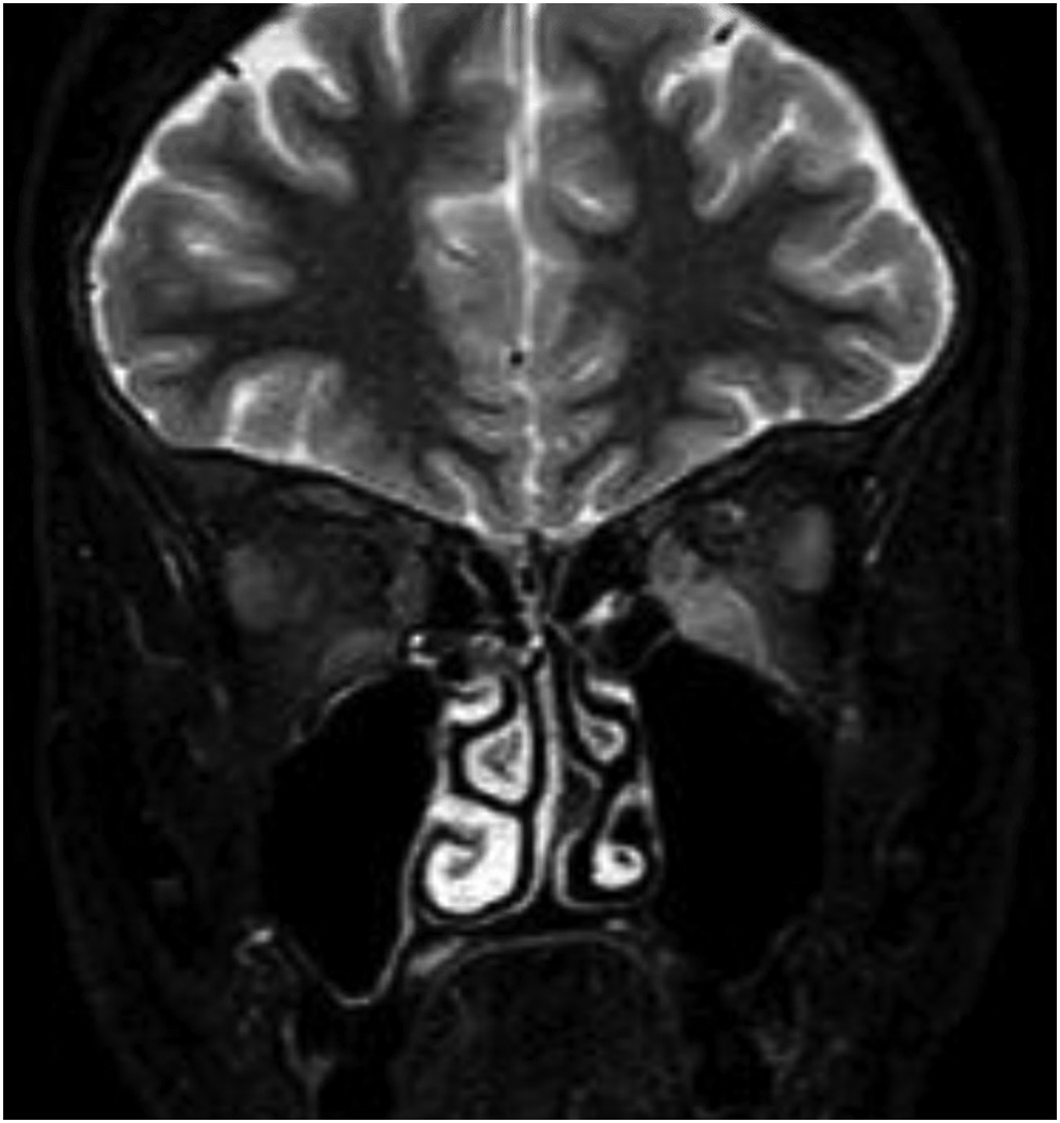
Initial MRI orbits STIR sequence coronal view; there is increased thickness of the inferior and medial rectus muscles bilaterally, particularly the right inferior rectus.

She was re-referred to the eye clinic due after 2-months from the endocrinologists due to suspected worsening retraction of the right upper lid. Examination showed slightly increased upper marginal reflex distance measurements of 8 mm right eye and 4.5 mm left eye. Repeated exophthalmometry remained stable with no proptosis and ocular motility was full, with no restriction. Initial thyroid function tests showed borderline low serum thyroid-stimulating hormone (TSH) of 0.24 mIU/L with normal free T4 and free T3 of 16 pmol/L and 4.3 pmol/L, respectively. Her TSH-receptor antibodies (TRAbs) were negative at <1.0 IU/L (Table 3). Subsequent serum TSH and free T3 and T4 were consistently within normal reference ranges (Table 3). Thyroid peroxidase antibodies and anti-thyroglobulin antibody were both negative. TRAbs were initially within the normal reference range, but during her final admission they became mildly positive at 2.3 IU/L (normal reference value: 1–1.8 IU/L) (Table 3). MRI orbits showed radiographic grade 1 proptosis with bilaterally increased thickness of inferior and medial rectus muscles; the most likely diagnosis was deemed TED (Fig. 2).

**Table 3:** Thyroid function tests and TRAb results.

	Normal range	February 2021	April 2021	October 2021
TSH (mIU/L)	0.34–4.5	0.44	3.17	2.81
Free T4 (pmol/L)	9–23	16	22	19
Free T3 (pmol/L)	3.5–6.6	4.3	5.4	4.1
TRAbs (mIU/L)	1–1.8	<1.0		2.1

TRAbs, thyroid receptor antibodies; TSH, thyroid-stimulating hormone.

Differential diagnoses included immunoglobulin G4-related disease (IgG4) and sarcoid-related ophthalmopathy; however, subsequent serum IgG4 and angiotensin-converting enzyme were both within normal limits. She was referred to a neuro-radiology multidisciplinary team at a tertiary unit where the consensus was that this patient had euthyroid eye disease.

**Figure 3:**
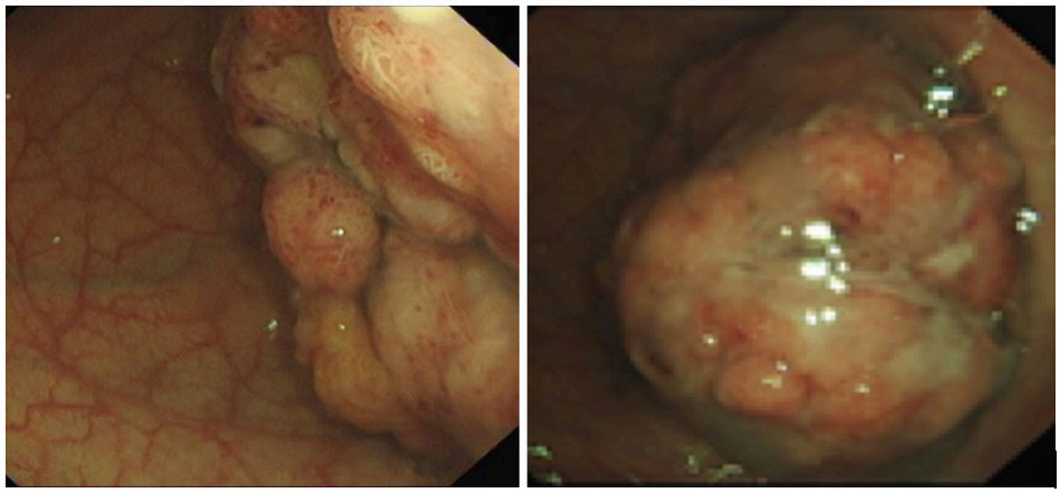
Flexible sigmoidoscopy; there is a large polypoidal lesion at the rectosigmoid junction.

By the fourth hospital admission, 8 months from initial presentation, the patient developed profuse rectal bleeding. This led the gastroenterology and endocrine specialists to request a contrast enhanced CT scan of the chest, abdomen and pelvis and flexible sigmoidoscopy. Findings revealed a large 8.5 cm recto sigmoid tumour with associated intussception (Fig. 3). Given the large size of the tumour, formal surgical excision was undertaken on that admission. The patient underwent a laparoscopic high anterior resection with a primary anastomosis. She had an uneventful post operative recovery. Histological analysis of the resected specimen was consistent with T1 N0 M0 adenocarcinoma within a large tubulovillous adenoma. The finding of a colonic tumour in the context of severe electrolyte derangement and acute kidney injury alerted the attending physicians to the rare diagnosis of MWS.

Following successful surgical resection of her tumour, the patients’ electrolyte imbalance, diarrhoea and eyelid retraction improved, with repeat MRI scan displaying complete resolution of rectus muscle enlargement and normal appearances of both orbits. The spontaneous improvement in EOME cemented the fact that this was a para neoplastic phenomenon. A completion colonoscopy performed 6 weeks post-operatively revealed no signs of recurrence or other polyps.

## Discussion

PS are uncommon with incompletely understood pathophysiology. One mechanism postulates that immune T cells synthesised to destroy neoplastic cells mistakenly target healthy tissue, which is known as molecular mimicry. Another mechanism sees the aberrant production or secretion of bioactive substances, electrolytes or hormones from neoplastic tissue which can induce a multitude of symptoms. PS are known to manifest in numerous bodily systems and can frequently masquerade as other clinical entities, including encephalitis, Cushing’s syndrome and polymyositis, to name a few. Ophthalmic related PS are well described in the literature, including cancer-associated retinopathy, para neoplastic villiform maculopathy and EOME (5, 6).

Gastrointestinal tumours commonly cause PS such as cachexia, acanthosis nigrans and dermatomyositis. There are case reports of gastrointestinal tumour causing PS orbital inflammation, particularly IgG4-related disease, but none of these specifically cause EOME in our knowledge. (7).

TRAbs, insulin-like growth factor and cytokines are elevated in autoimmune thyroid disease. They interact with the orbital fibroblasts, leading to orbital inflammation which causes orbital signs like proptosis and eye lid retraction. Similarly, cytokines and hormones released from the neoplastic tumours, e.g colonic cancer as a para neoplastic phenomenon, cause similar orbital changes. In addition to the well-known effect of TRAbs on orbital fibroblasts, studies speculate their tumour-promoting role in carcinogenesis (8).

Endocrine- and renal- related PS are rare in gastrointestinal tumours but can precede the tumour itself with deranged biochemistry and vague constitutional upset, leading to a delay in diagnosis of the primary neoplasm. MWS due to distal colonic tumours often has an insidious natural history. The most extensive systematic review of this clinical entity found a total of only 257 cases in the literature reported across all languages (4). Key features characterising MWS include profuse watery or mucoid diarrhoea, acute kidney injury and severe hyponatraemia and hypokalaemia. The proposed pathophysiology is a hypersecretory loss of fluid, possibly more than 4.0 L/day, and electrolytes through the tumour into the gastrointestinal tract (9). Significant mortality is associated with this hypersecretory tumour and subsequent excisional surgery, with overall mortality reported to be around 10.5% in MWS (4). Our patient also developed secondary hyperaldosteronism secondary to severe and persistent diarrhoea and hypokalaemia, which is usually expected due to volume depletion.

Aggressive fluid resuscitation often temporarily resolves symptoms and electrolyte disturbances, which is why patients can re-present multiple times (as was the case with our patient) before a definitive diagnosis is made. Once a tumour is detected in such cases, it is essential for an experienced colorectal team to manage these patients as frequently MWS tumours are large and distal in the gastrointestinal tract. Major surgery is usually required, with traditional anterior or abdominoperineal resective approaches occurring most commonly, which was the case in this patient

The clinical manifestations of TED are eyelid retraction, retrobulbar pain, diplopia, periorbital oedema and inflammation and proptosis, which are commonly bilateral (10). Orbital MRI is an adjunctive investigation to clinical ophthalmic assessment to further characterise the severity of TED and to rule out other differential diagnoses in unusual presentations, including unilateral orbital signs or in patients who are euthyroid with negative thyroid autoantibodies (11). Typical radiological findings of TED include proptosis, adipose tissue expansion and hypertrophy of multiple extra-ocular muscles without tendon involvement, with the inferior rectus typically the earliest muscle affected followed by the medial rectus (12). A small proportion of patients with TED, approximately 5%, are euthyroid, and around 30% of these euthyroid patients also have negative thyroid autoantibodies (13). The biopsy of the orbital muscle does help to substantiate the pathogenesis as in this quoted case (14) where histology demonstrated lymphatic infiltration of the orbital muscle leading to the diagnosis. However, in our case, biopsy of the orbital muscle was not performed. Our case highlights the importance of considering other differential diagnoses when presented with a patient who appears to have euthyroid ophthalmopathy.

According to Rundle's curve, Graves' ophthalmopathy (GO) worsens during an initial phase up to a peak of maximum severity and then improves and reaches a static plateau. Several studies confirm that untreated, mild GO undergoes a spontaneous improvement in a substantial proportion of patients. It remains unchanged almost in a similar number of patients, and in a minority of them it worsens (15). In our case, untreated, mild GO worsened initally and then remained stable.

To our knowledge, this is the first report of a PS-EOME caused by a primary colorectal malignancy. This highlights another rare feature that can be associated with the already rare diagnosis of MWS. In our patient, the diagnosis was difficult to ascertain; the radiographic pattern and character of EOME was similar to that of TED, as well as lid retraction and diarrhoea bolstering this differential. However, the diagnosis was reached through prompt and effective collaboration between gastroenterologists, endocrinologists and ophthalmologists, and successful surgical treatment led to a good outcome.

## Declaration of interest

The authors declare that there is no conflict of interest that could be perceived as prejudicing the impartiality of the research reported.

## Funding

This research did not receive any specific grant from any funding agency in the public, commercial or not-for-profit sector.

## Patient consent

Written informed consent for publication of their clinical details and clinical images was obtained from the patient.

## Author contribution statement

W Ahmad is the main Endocrinologist Physician who saw the patient initially then followed the patient and also reviewed and edited the manuscript. M Hartley and K Motohashi conducted the literature review and manuscript writing. H Dallal was responsible for the gastroenterology care of the patient and provided with the Fig. 3 of the case. D Kamali performed the surgery. C Matthews and S Singh were responsible for the ophthalmology care and provided with Figures 1 and 2 of the case. S Kamaruddin and S A Tee contributed towards patient care as part of the Department of Endocrinology.
